# Mineral-Targeted Microbial Enhanced Oil Recovery

**DOI:** 10.3390/microorganisms13122706

**Published:** 2025-11-27

**Authors:** Lei Li, Chunhui Zhang, Peidong Su

**Affiliations:** School of Chemical & Environmental Engineering, China University of Mining & Technology (Beijing), Beijing 100083, China

**Keywords:** microbial enhanced oil recovery, mineral-targeted, fluid-microbe–mineral ternary system, microbial weathering, microbial mineralization, microbial leaching

## Abstract

In the fluid (comprising oil and nutrient solution)–microbe–mineral ternary system of oil reservoirs, current microbial enhanced oil recovery (MEOR) technology lacks investigation into the interactions between the latter two components and their application potential in petroleum production. This may explain why MEOR has achieved only partial success while failing to meet full expectations. This review systematically synthesizes the existing fragmented research on reservoirs regarding rock minerals as direct/indirect microbial substrates in MEOR applications. Currently, microbe–mineral interactions enhance oil recovery primarily through the following mechanisms: clay swelling inhibition, induced mineral precipitation, silicate dissolution, wettability alteration, microbial acids etching, and hydrocarbon degradation modulation. Integrating contemporary findings on microbe–mineral interactions, three strategically prioritized MEOR implementation pathways demonstrate particular promise: microbially mediated weathering processes in silicate/carbonate reservoirs, microbial-induced mineral precipitation/dissolution cycles, and microbial leaching-assisted permeability enhancement. Finally, a total of 20 microorganisms potentially applicable for mineral-targeted MEOR were proposed. If MEOR technology could be re-examined from the perspective of microbe–mineral interactions and thoroughly investigated, integrating the knowledge on fluid–microbe binary systems in oil reservoir, this potentially transformative technology may achieve breakthroughs.

## 1. Introduction

Despite significant efforts in developing and utilizing new energy sources, the world still heavily relies on petroleum resources. As an indispensable strategic resource for modern societal development, crude oil plays a pivotal role in global industrial operations, economic propulsion, and innovation-driven growth. In developed oil reservoirs, approximately two-thirds [1,2,3,4,5] of crude oil remains unrecovered after primary (flowing production [6,7]) and secondary recovery (waterflooding) processes. To access these considerable reserves, almost all available technical methods have been attempted, including physical (ultrasonic [8,9] and electrokinetic [10]); thermal (steam [11,12], hot water [13], in situ combustion [14], and combined thermo-chemical [15,16]); chemical (surfactant [17], polymer [18], alkaline [19], and solvent [20]); gas (CO_2_ [21] and air [22]); and microbial methods. Most enhanced oil recovery methods that involve energy and mass transfer inevitably imply high costs and substantial pollution. However, microbial enhanced oil recovery (MEOR) technology does not require the continuous input of energy or materials, typically requires minimal surface and downhole equipment support, and causes minimal contamination to both surface and subsurface environments. Consequently, it is widely acknowledged for its dual advantages of economic feasibility and environmental sustainability, garnering significant attention from researchers [3,23].

**Figure 1:**
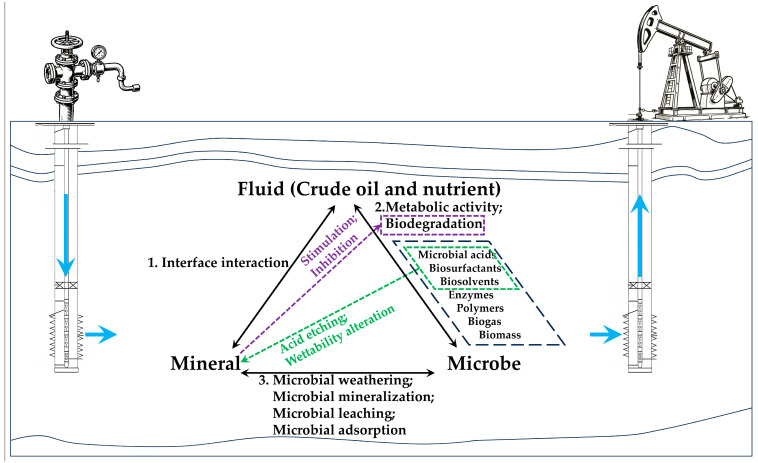
Schematic diagram of the interactions of the fluid–microbe–mineral (FMM) ternary system in MEOR. Note: The blue arrows indicate the directions of injection and production.

Traditionally, MEOR has been defined as a tertiary oil recovery method that utilizes microorganisms and their metabolites—such as biomass, biogas [24], biosurfactants [25], microbial acids [26], biosolvents [27,28], and enzymes [29]. Since these metabolic products are typically generated by the microbial metabolism of crude oil [30] or nutrients [31], it is easy to overlook the interactions between the latter two components (microbe and mineral) within the fluid–microbe–mineral (FMM) ternary system in oil reservoirs (Figure 1). This, however, may well explain why despite the substantial potential of MEOR technology being widely acknowledged, its industrial applications have yet to deliver a consistently satisfactory performance [1,27,32].

This review provides the first systematic analysis of MEOR mechanisms involving both direct and indirect microbial interactions with rock minerals. Direct microbial actions include microbial biomineralization that inhibits clay swelling [33,34], microbially induced precipitation for plugging high-permeability channels [35,36], and silicate-dissolving microorganisms directly acting on silicate minerals to enhance permeability [37]. Indirect effects refer to the influence of microbial metabolites (biosurfactants [38], biosolvents [39,40,41,42], and microbial acids [27]) generated through fluid–microbe interactions on rock minerals. In reality, microbe–mineral interactions occur more extensively through direct mechanisms than via indirect reactions mediated by metabolic byproducts in most geological contexts [43,44,45]. Furthermore, this review proposes potentially the most promising mineral-targeted MEOR development directions. The proposed research framework focuses on investigations within the FMM system under reservoir conditions (including but not limited to temperature, pressure, and material composition), specifically encompassing the microbial weathering of silicate and carbonate minerals [46], microbial mineralization [47,48], and microbial leaching [49,50,51]. This provides the field with a previously neglected perspective that serves to review and restructure both MEOR research and its applications.

## 2. MEOR

The earliest conceptualization of MEOR was proposed by Beckmann in 1926 [52]. It was not until the 1940s that researchers like Zobell et al. initiated experimentally validating this concept [53]. The first field-scale implementation of MEOR occurred at the Lisbon Oil Field in Union County, Arkansas, USA [54]. Lazar conducted a systematic review of 30 pre-1991 MEOR field implementations [55], while Maudgalya subsequently documented and analyzed 407 globally distributed MEOR field applications prior to 2007 [1]. In addition, based on summaries of MEOR field tests conducted in China [56], USA, Russia, and other countries [57,58], six primary application types have been identified: microbial flooding recovery [59], cycle microbial recovery [56], microbial selective plugging recovery [54], microbial wax removal [60], genetically engineered microbial enhanced oil recovery [61], and enzyme enhanced oil recovery [29]. The majority of them are generally considered successful. However, few of the tests explain the mechanics of the oil recovery or presented post-treatment analyses or how the results were calculated. This helps explain why MEOR has not gained credibility in the oil industry [1,32].

The mechanisms of MEOR mediated by microbial metabolites can be systematically categorized into four principal components: (1) Biosurfactants predominantly function to diminish oil/water interfacial tension, alter the wettability of porous media, emulsify residual oil, and enhance bacterial migratory capacity [62,63]. (2) Biopolymers, biofilms, and microorganisms selectively occlude high-permeability porous media, wherein biopolymers additionally serve as viscosity modifiers to augment aqueous phase viscosity [56,64]. (3) Biogenic gases, solvents, and acids facilitate the dissolution of carbonate reservoir rocks, thereby enabling aqueous phase penetration into rock pore systems for enhanced residual oil contact. Concurrently, the liberated gases from carbonate dissolution contribute to reservoir pressure augmentation [65]. (4) Reservoir-residing microorganisms utilize crude oil as a carbon substrate, metabolizing long-chain saturated hydrocarbons to reduce crude oil viscosity and improve its mobility [66].

With the rapid advancement of biotechnology, novel functional microbial strains are continuously isolated, identified, and validated for their efficacy in enhancing oil recovery [67,68,69]. The increasing maturity of genetic engineering techniques has enabled the optimization of microbial performance [70,71]. Research on reservoir microorganisms has yielded significant breakthroughs. Comprehensive documentation exists regarding microbial community dynamics across various reservoir types, operational parameters, and temperature–pressure conditions [72,73,74,75].

Nevertheless, it can be empirically concluded that current MEOR research remains fundamentally a black-box paradigm [76,77]. The underlying interaction mechanisms among multifactorial components are yet to be systematically deciphered. Given the substantial body of research on fluid–microbe interactions in MEOR, microbe–mineral interactions under reservoir conditions likely constitute the critical knowledge gap in our understanding.

## 3. Microbial Inhibition of Clay Swelling

**Figure 2:**
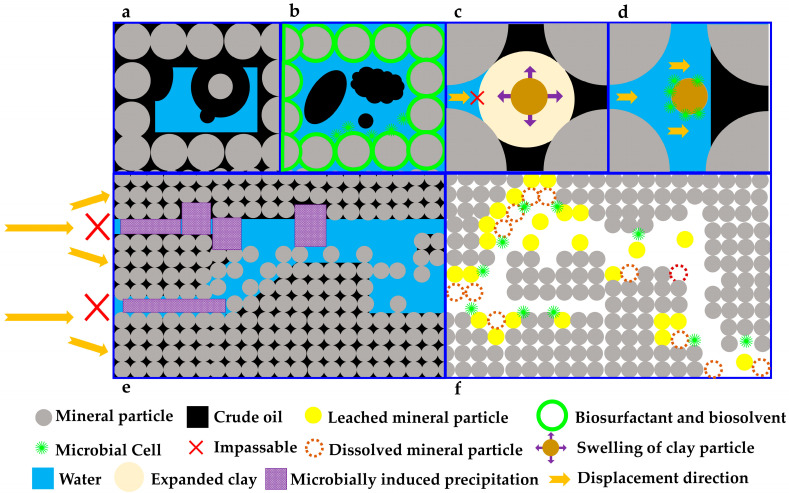
Schematic diagram of the mechanism for microbial enhanced oil recovery through microbe–mineral interactions in the fluid–microbe–mineral ternary system of oil reservoirs. Note: (**a**) In the initial state, the rock mineral surfaces in the reservoir are oil-wet, with crude oil trapped on them and predominantly distributed in pore corners, while water exists as a free phase. (**b**) Under the influence of biosurfactants, biosolvents, and microbial cells, the wettability of the mineral surfaces is altered, leading to a reversal of oil and water distribution compared with (**a**). (**c**) Swelling of clay minerals blocks the flow pathways of the displacing fluid (typically water), preventing the crude oil in the pores from being produced. (**d**) Microbial activity effectively suppresses clay swelling, ensuring unimpeded flow channels and enabling the displacement of crude oil from the pores. (**e**) Microbially induced precipitation blocks high-permeability water flow channels, diverting the displacing phase into previously unswept areas and mobilizing the crude oil therein. (**f**) Through microbial weathering—either acid-mediated or non-acidic—mineral particles are dissolved, increasing the population of leached mineral particles and thereby enhancing reservoir porosity and permeability.

Reservoirs commonly contain clay minerals, among which swelling clays (e.g., montmorillonite, beidellite, and saponite) exhibit dramatic hydration swelling (volumetric increases of 600–1000%) upon water contact (with formation water or injected water) [78]. Clay swelling significantly reduces reservoir porosity, permeability, and oil recovery efficiency [79] (Figure 2), particularly in low-permeability and ultralow-permeability reservoirs [33]. Therefore, effective strategies should be implemented during reservoir development to mitigate clay swelling, thereby enhancing reservoir permeability and hydrocarbon production (Table 1).

**Table 1:** Mineral-targeted MEOR studies.

No.	Year	Type	Microorganism	Mechanism	Result	Ref.
1	2013	Laboratory experiment	*Enterobacte* *r* *cloacae*	Biosurfactants alter mineral wettability	Increased crude oil recovery by 5% to 10%	[41]
2	2016	Field test	*Sporosarcina* *pasteurii*	Microbially induced calcium carbonate precipitation	Pressure rose, injection rate dropped (from 1.9 to 0.47 L/min)	[80]
3	2017	Laboratory experiment	Microbial communities	Microbial acids-induced carbonate dissolution	Porosity increased by 14.89–68.29%, and permeability improved by 35.77–137.83%	[81]
4	2018	Field test	*Sporosarcina* *pasteurii*	Microbially induced calcium carbonate precipitation	Injection rate dropped (1.28 gallons per minute (gpm) to less than 0.05 gpm)	[82]
5	2018	Laboratory experiment	4 Fe(III)-reducing microbial strains	Inhibition of montmorillonite hydro-swelling	Inhibition of Ca-montmorillonite swelling at a rate of 48.9%	[33]
6	2018	Laboratory experiment	*Alcaligenes* *faecalis*	Biosurfactants alter mineral wettability	Contact angle decreased from 156° to 86°, shifting from oil-wet to intermediate-wet, enhancing oil recovery by 5.2–8.2%	[42]
7	2019	Laboratory experiment	*Bacillus* *subtilis*	Biosurfactants alter mineral wettability	The wettability was modified from the values indicating an intermediate water-wet condition to a strong water-wet condition	[39]
8	2020	Laboratory experiment	*Sporosarcina* *pasteurii*	Microbially induced calcium carbonate precipitation	The permeability of large-, medium-, and small-aperture core samples declined to 47%, 32%, and 16% of their initial values, respectively	[83]
9	2022	Laboratory experiment	*Proteus* *hauseri*	Inhibition of montmorillonite hydro-swelling	The waterflooding injection pressure was reduced by 61.1%, while the core permeability and oil recovery were enhanced by 49.6% and 8.1%, respectively	[84]
10	2023	Laboratory experiment	*Flaviflexus* *huanghaiensis,* *Shewanella* *chilikensis*	Inhibition of hydro-swelling and prevention of plugging-related damage	The relative anti-swelling rate of montmorillonite in water improved by 46.2%, 39.7%, 36.6%, 38.4%, and 34.6% under different pressures	[34]
11	2023	Laboratory experiment	*Acidithiobacillus thiooxidans, Acidithiobacillus ferrooxidans, Sulfobacillus thermosulfidooxidans*	Biosurfactants alter mineral wettability	Microorganisms promoted a highly water-wet condition but enhanced asphaltene adsorption	[40,85]
12	2025	Laboratory experiment	*Paenibacillus* *mucilaginosus*	Microbial-mediated crystallization	Core permeability decreased by 66.67%, the porosity dropped to 8.32%, the plugging rate reached 63.08%	[35]
13	2025	Laboratory experiment	*Paenibacillus mucilaginosus*	Dissolution of silicate minerals under neutral conditions	The porosity increased by 1.4% and permeability increased by 12.3 mD of low-permeability cores	[37]
14	2025	Laboratory experiment	*Bacillus* *subtilis*	Prevention of asphaltene adsorption on carbonate minerals	The bioproducts reduced the asphaltene adsorption by up to 75%	[86]

Research has demonstrated that indigenous Fe(III)-reducing microbes [84], such as *Shewanella* [87], *Bacillus* [88], *Deferribacter thermophilus* [89], and *Geoalkalibacter subterraneus* [90], exhibit high environmental adaptability to oil reservoirs and can mediate structural transformation. These microbes facilitate the transformation of swelling montmorillonite into non-swelling illite or other secondary minerals, thereby effectively suppressing clay swelling oil reservoirs.

Dissimilatory iron-reducing bacteria (DIRB)-mediated bioreduction can also significantly alter the physicochemical properties of clay minerals, thereby accelerating the illitization of montmorillonite [34,91]. The structural destabilization of montmorillonite induced by DIRB-driven bioreduction leads to the degradation of its crystalline framework, accompanied by the release of structural and interlayer water molecules. Consequently, this process markedly diminishes both interlayer and external swelling capacity, while facilitating the contraction of montmorillonite [92].

Water intrusion into hydrocarbon reservoirs containing swelling clays appears inevitable during both drilling and development phases, making induced water-sensitive formation damage one of the key factors contributing to productivity decline. The early intervention of microbial clay swelling prevention techniques, as early as the initial stages of hydrocarbon development or even during drilling operations, represents a highly promising solution strategy. Building upon preventive measures, research on microbial-induced clay contraction holds even greater value for reservoirs already affected by water-sensitive damage.

## 4. Microbially Induced Precipitation

Reservoir heterogeneity adversely impacts sweep efficiency during waterflooding, as injected fluids preferentially migrate through high-permeability “thief zones”, bypassing oil-saturated low-permeability regions. The selective plugging of these “thief zones” forcibly redirects displacement fluids into previously unswept, oil-bearing zones [83] (Figure 2). This flow redistribution equilibrates volumetric flux between high- and low-permeability strata, resulting in a more uniform flood front advancement and significantly improved macroscopic sweep efficiency. Consequently, utilizing MEOR for targeted “thief zones” conformance control emerges as the most technically viable, operationally practical, and economically feasible method to optimize sweep efficiency and enhance ultimate hydrocarbon recovery.

One of the most prevalent occurrences involves microbially induced carbonate precipitation (MICP) [83]. *Sporosarcina pasteurii*, the most widely utilized microorganism in MICP applications, secretes highly active urease during its metabolic processes [93,94] (Table 1). This enzyme catalyzes the hydrolysis of urea into ammonia (NH_3_) and carbon dioxide (CO_2_) (Figure S1). The resultant hydrolytic products subsequently undergo diffusional transport across the cell envelope into the bulk aqueous phase, where rapid secondary hydrolysis generates ammonium cations (NH_4_^+^) and carbonate anions (CO_3_^2−^). Under conditions of local calcium ion (Ca^2+^) supersaturation, these carbonate moieties participate in heterogeneous nucleation via ionic association, ultimately precipitating as crystalline calcium carbonate (CaCO_3_) [95,96] (Figure S1). A recent study has demonstrated that *Paenibacillus mucilaginosus* can mediate CO_2_ fixation into amorphous and crystalline carbonate minerals. This process not only contributes to MEOR but also provides novel insights for carbon sequestration strategies [35]. Furthermore, the plugging capacity of *Bacillus subtilis*-mediated MICP has been extensively characterized [97]. Beyond single microbial strains, studies have demonstrated that microbial consortia-mediated MICP exhibits a superior performance—a finding that warrants greater attention in MEOR applications [98].

Of note, any form of controlled microbial mineralization (microbially induced crystallization phenomenon) in reservoir formations holds potential for MEOR applications. This encompasses the precipitation of calcite, aragonite, vaterite, and dolomite [99,100,101]. Particularly, inorganic phosphate precipitation may demonstrate the most significant potential for MEOR implementation. Microorganisms facilitate inorganic phosphate precipitation either by promoting direct sedimentation or through cellular assimilation into organic components [102]. Their primary roles involve supplying reactive phosphate/calcium phosphate, while maintaining pH and redox conditions conducive to phosphate precipitation. By modulating the precipitation–dissolution dynamics of these minerals, this approach not only accomplishes MEOR’s primary goal of microbial conformance control in high-permeability zones, but also delivers auxiliary benefits: the released mineral ions stimulate metabolic activity in other MEOR functional microbial communities. Notably, the petroleum industry exercises stringent control over the formation of all sulfur-bearing minerals that may ultimately convert to hydrogen sulfide, given its severe threats to operational safety.

## 5. Microbial Weathering of Silicates and Carbonate Minerals

Globally, carbonate reservoirs have emerged as primary hydrocarbon production resources owing to their widespread distribution, consistent thickness, and extensive scale. The Middle East contributes nearly two-thirds of global oil output, with 80% of its oil-bearing formations comprising carbonate rocks. In North America, approximately half of the total oil production is derived from carbonate reservoirs. China hosts nearly 3 × 10^6^ km^2^ of carbonate rocks, covering roughly one-third of its terrestrial area [103]. Silicate minerals such as feldspar, clay, quartz, and mica are also widely present in oil reservoirs [104]. Among these, clay minerals often act as cementing agents and interstitial fillings between mineral grains [105,106], and their disruption may lead to particle detachment (Figure 2). As the two dominant rock mineral types in reservoirs, research findings on their microbial weathering could likely enhance the permeability of rock media in reservoirs, ultimately achieving the goal of improved oil recovery.

Silicate-dissolving bacteria demonstrate robust capabilities in decomposing silicate minerals under neutral pH conditions [107,108,109,110]. Traditionally, these bacteria have been extensively utilized in agricultural biofertilizers, where they mobilize potassium ions from silicate minerals for crop nutrition [111]. In a groundbreaking application, researchers have pioneered their use in MEOR [37] (Table 1). Comparative core flooding experiments revealed that *Paenibacillus mucilaginosus* significantly enhances pore network connectivity (porosity increase by 1.4%) and fluid transport capacity (permeability improvement by 12.3 mD) under neutral pH conditions, ultimately achieving a 6.9% incremental oil recovery factor [37] (Table 1 and Figure 2). It is foreseeable that other microorganisms co-applied with silicate-dissolving bacteria in MEOR may utilize their silicate-derived metabolic byproducts as nutrients, creating a synergistic promotion effect.

Although the role of microbial acids etching in enhancing reservoir rock permeability is widely acknowledged, dedicated studies on this topic remain scarce (Table 1). Most research only peripherally mentions this potential MEOR mechanism during efficacy analysis [112,113,114], likely because microbial acids corrosion is generally presumed to have a limited impact on substantial oil recovery improvement. This explains why reports on acid-producing microorganisms, biogenic acid characteristics, and their effects on the porosity and permeability of oil reservoir remain scarce [115]. A comparative investigation has revealed that acid-producing bacterium (*Bacillus licheniformis*) alone exhibits inferior oil recovery enhancement compared with biosurfactant-producing bacterium (*Pseudomonas aeruginosa*) or even silicate-dissolving bacterium (*Paenibacillus mucilaginosus*) [37]. Another separate study, directly relating rock type to MEOR and pH buffering, reports the result of an experimental study conducted using microbial communities from an oil reservoir with low-permeability (<40 mD) limestone rock samples [81]. The post-MEOR treatment analysis of four replicate core samples revealed an average increase in porosity and permeability. Notably, the study recorded a significant pH decrease from neutral (7.0) to acidic conditions (5.2 ± 0.5) [81] (Table 1).

The microbial weathering of minerals is a synergistic effect driven by multiple factors, including but not limited to organic acid dissolution [116,117], redox reactions [118,119], chelation [120,121], and biomechanical processes [122]. Microbially produced organic acids comprise bacterial acids (formic, acetic, lactic, pyruvic, succinic) and fungal acids (gluconic, oxalic, citric). These organic acids not only significantly reduce the pH of local microenvironments but also chelate metal cations in minerals. For example, dihydroxybenzoic acid and salicylic acid can chelate aluminum, iron, and calcium ions [123]. Chelation occurs between microbial-derived biomacromolecules/polymers and mineral elements [124], which is essentially a mineral solubilization process [125]. As microorganisms can use insoluble minerals as electron acceptors [126] to drive redox reactions, the redox transformation of compounds within mineral structures destabilizes the crystal lattice. Consequently, the mineral structure is disrupted, potentially leading to dissolution [127]. Studies have also shown that quinones, cysteine, and melanin-like heteropolymers may directly participate in electron transfer with minerals [128,129] and potentially contribute to microbial mineral weathering.

If relying solely on microbial weathering alone, its relatively low reaction rate prevents the immediate enhancement of petroleum production efficiency and crude oil recovery. However, over longer time scales, its impact may become non-negligible. For instance, applying such microbes in production reservoirs scheduled for long-term shutdown—where no additional operations are conducted during the closure period—could yield gradual positive effects. Unfortunately, no studies have yet reported on such subtle yet beneficial measures.

## 6. Wettability Alteration

Wettability analysis serves as a fundamental parameter in reservoir engineering, governing capillary pressure dynamics, irreducible water saturation, residual oil saturation, and ultimate oil recovery rates [130,131]. Research has demonstrated that the majority of carbonate reservoirs display oil-wet behavior, owing to the chemisorption of polar organic compounds (particularly asphaltenes and naphthenic acids) onto the carbonate mineral surfaces [132,133] (Figure 2). Surfactant adsorption efficiency in subsurface formations is predominantly controlled by rock mineralogy, which ultimately dictates the degree of wettability modification [134].

Biosurfactants and biosolvents derived from microbial metabolism actively modify the wettability of rock minerals during MEOR processes [39] (Table 1 and Figure 2). Multiple microbial strains, particularly those belonging to the genera *Bacillus*, *Rhodococcus*, *Acinetobacter*, *Enterobacter*, *Alcaligenes*, and *Pseudomonas*, exhibit efficient biosurfactant production in MEOR [39,41,42,135,136,137] (Table 1). Bacterial adhesion and biofilm formation constitute another key wettability alteration mechanism (Figure 2). Cells preferentially colonize surfaces rather than proliferating planktonically in aqueous media [138]. Biofilms show significantly higher antimicrobial resistance than planktonic cells. Bacterial adhesion and biofilm formation induce physicochemical modifications at rock surfaces. While the exact wettability alteration mechanisms require further elucidation, the predominant hypotheses (that include bacterial surface adhesion, biofilm formation, the adsorption of bacterial metabolites, and biosurfactant activity [138]) indicate that the shift from oil-wet to water-wet rock surfaces facilitates the displacement of crude oil from reservoir pores.

Biosurfactants and biosolvents capable of altering the wettability of mineral surfaces, though originating from fluid–microbe interactions, ultimately target minerals and thus qualify as indirect microbial interactions with rock minerals. In contrast, the direct adhesion of bacterial cells to mineral surfaces constitutes direct interactions. In reality, both microorganisms and their metabolic products (e.g., surfactants and organic solvents) tend to accumulate predominantly at the oil–water interface rather than the oil–rock interface. This preference arises because the oil–water interface offers richer nutrients, gases, and aqueous environments, whereas penetrating the viscous crude oil to reach the oil–rock interface requires greater propulsion capacity and leads to inferior metabolic conditions for most microorganisms. Only specific microbes—such as lithophilic bacteria, endolithic bacteria, and mineral-colonizing bacteria—possess the capability to effectively colonize mineral surfaces, thereby enabling the more efficient alteration of mineral surface wettability.

## 7. Impact of Minerals on Microbial Hydrocarbon Degradation

The minerals present in oil reservoirs (feldspar, quartz, calcite, kaolinite, illite, smectite, and chlorite) influence crude oil degradation. Although the underlying mechanisms have rarely been studied in detail [139,140], this mineral-driven effect likely serves as a pivotal mechanism in MEOR processes.

The research findings in this field demonstrate that different minerals exert distinct effects on microbial hydrocarbon degradation. A comparative study on four iron-bearing mineral phases demonstrated that magnetite, hematite, and ferrihydrite significantly enhance hydrocarbon biodegradation, whereas Fe^3+^ exhibited inhibitory effects [141]. These were mainly attributed to the reinforced interspecific relationships induced by special species and the synergistic effects of substance conversion under the biocurrent stimulation [141].

The influence of clay minerals on microbial hydrocarbon degradation is highly complex (Table 2). For example, although illite exerted a negative effect on *Pseudomonas stutzeri* degrading heavy oil by inhibiting the biodegradation of 64 saturated hydrocarbons and 50 aromatic hydrocarbons, it selectively stimulated the biodegradation of 45 aromatic hydrocarbons with a specific structure [142]. As another example, despite reports of kaolinite’s inhibitory effects on microbial hydrocarbon degradation, a number of studies have documented its capacity to stimulate the process (Table 2). As a third example, saponite enhances microbial hydrocarbon degradation in some studies, but shows no effects (either positive or negative) in others (Table 2).

In addition, most clay minerals such as montmorillonite, palygorskite, vermiculite, bentonite [143], and nontronite [144] consistently demonstrate stimulatory effects on microbial hydrocarbon degradation (Table 2). The observed variations may result from differences in microbial conditions (species or consortia), crude oil composition (saturated hydrocarbons, aromatic hydrocarbons, resins, and asphaltenes; SARA fractions), compound-specific responses (phenanthrene, naphthalene, and anthracene), or the source of clay minerals [142] (Table 2).

The microbial degradation of crude oil generates metabolites utilized in MEOR, and the influence of minerals on this process undoubtedly exerts indirect effects on ultimate recovery efficiency. However, current research findings predominantly focus on the microbial remediation of petroleum contamination, representing typical surface environments (e.g., soil and water bodies). Studies examining mineral impacts on microbial crude oil degradation under authentic reservoir conditions (e.g., temperature, pressure) remain relatively limited.

**Table 2:** Effect of minerals on the microbial degradation of petroleum hydrocarbons.

No.	Mineral	Substrate	Effect	Mechanism of Influence	Degrader	Ref.
1	Mix clay	Crude oil	Stimulation for saturated hydrocarbons, neutral for aromatic hydrocarbons	Increases biological accessibility	Microbial community	[145]
2	Kaolinite	Heavy oil in the environment	Stimulation	C-O-Na-Si stimulates metabolism	Microbial community	[146]
3	Montmorillonite	Heavy oil in the environment	Stimulation	Stimulates growth and buffer pH	*Pseudomonas aeruginosa* + Microbial community	[147]
	Kaolinite					
4	Montmorillonite	Heavy oil in the environment	Stimulation	Stimulates growth and buffer pH, C-O-Na-Si stimulates metabolism	Microbial community	[148]
	Montmorillonite		Stimulation			
5	Vermiculite	Naphthalene, Anthracene	Stimulation	Protects from toxicity	Microbial community	[149]
6	Montmorillonite	Crude oil	Stimulation	Adsorbent	Microbial community	[150]
7	Montmorillonite	Saturated hydrocarbons in crude oil	Stimulation	High specific surface area	Microbial community	[151]
	Palygorskite		Stimulation	High specific surface area		
	Saponite		Neutral	/		
	Kaolinite		Inhibition	No local bridging effect, low specific surface area		
8	Saponite	Crude oil	Stimulation	High specific surface area and cation exchange capacity	Microbial community	[152]
9	Kaolinite	Crude oil	Inhibition	Low specific surface area and cation exchange capacity	Microbial community	[153]
	Palygorskite		Stimulation	High specific surface area and cation exchange capacity		
	Saponite		Neutral	/		
	Montmorillonite		Stimulation	High specific surface area and cation exchange capacity		
10	Montmorillonite	Crude oil	Stimulation	/	Microbial community	[154]
11	Montmorillonite	Phenanthrene and dibenzothiophene compounds	Stimulation	/	Microbial community	[155]
12	Calcium bentonite	Crude oil in the environment	Stimulation	High specific surface area	Microbial community	[156]
	Fuller soil		Stimulation			
	Kaolinite		Stimulation			
13	Palygorskite	Phenanthrene(C^14^)	Stimulation	Stimulate biofilm formation and accommodate extracellular enzymes	*Burkholderia sartisoli*	[157]
14	Montmorillonite	Phenanthrene(C^14^)	Stimulation	High specific surface area and cation exchange capacity	*Burkholderia sartisoli* + Microbial community	[158]
	Palygorskite					
15	Montmorillonite	Crude oil	Stimulation	Stimulates contact with nutrients	Microbial community	[159]
	Saponite		Stimulation	Increases nutrients utilization		
16	Montmorillonite	Aromatic hydrocarbons in crude oil	Stimulation	High specific surface area and cation exchange capacity	Microbial community	[160]
	Saponite		Stimulation			
	Palygorskite		Stimulation	Channel structure		
	Kaolinite		Inhibition	Influence of impurities		
17	Kaolinite	Phenanthrene	Stimulation	Silicon/oxygen atoms stimulate biological effects	*Sphingomonas* sp. GY2B	[161]
	Quartz		Stimulation			
18	Nontronite	Crude oil	Stimulation	Stimulates ion exchange and nutrient absorption	*Alcanivorax borkumensis*	[144]
19	Bentonite	Aromatic hydrocarbons and cadmium contaminated soil	Stimulation	Adsorption of heavy metals	Microbial community	[162]
20	Palygorskite	Crude oil contaminated soil	Neutral	/	Microbial community	[163]
21	Illite	Heavy oil	Inhibition for all saturated hydrocarbons and 50 aromatic hydrocarbons, stimulation for 45 aromatic hydrocarbons	Adsorption and cation-π	*Pseudomonas stutzeri*	[142]

Note: / indicates that there is no relevant information in the literature.

## 8. Promising Mineral-Targeting Microbes for MEOR

Although the current knowledge of microbe–mineral interplay within the FMM ternary system in MEOR remains incomplete, existing studies have confirmed their considerable potential for MEOR applications. Building upon the research findings on microbe–mineral interactions under non-reservoir conditions (non-MEOR targets), this review proposes that the microorganisms most likely to achieve breakthroughs in mineral-targeted MEOR in the future include lithophilic bacteria, endolithic bacteria, and mineral-colonizing bacteria, which demonstrate multifunctional capabilities encompassing, but not limited to, microbial weathering, mineralization, and leaching activities (Table 3).

**Table 3:** Promising mineral-targeting microbes for MEOR.

No.	Microorganism	Function	Ref.
1	*Acidithiobacillus ferrooxidans*	Microbial leaching of copper	[164]
2	*Acidithiobacillus thiooxidans*	Microbial leaching of chalcopyrite	[165]
3	*Arthrobacter* sp.	Accelerating the release of Fe from hornblende	[166]
4	*Bacillus cereus*	Dissolution of manganese	[167]
5	*Cupriavidus metallidurans*	Microbial leaching of copper	[168]
6	*Delftia acidovorans*	Formation of gold nanoparticles	[169]
7	*Ferroplasma acidarmanus*	Microbial leaching of pyrite, marcasite, and arsenopyrite	[170]
8	*Gallionella ferruginea*	Its organic molecules retard mineral growth	[171]
9	*Geobacter sulfurreducens*	Formation of Cr(III) crystals	[172]
10	*Leptospirillum ferrooxidans*	Microbial leaching of copper	[173]
11	*Leptothrix discophora*	Formation of ferromanganese nodules	[174]
12	*Mariprofundus ferrooxydans*	Co-precipitation with iron	[175]
13	*Methanocaldococcus jannaschii*	Metal ion binding-mediated silicification	[176]
14	*Nitrobacter winogradskyi*	Microbial weathering of serpentinized ultrabasites	[177]
15	*Pseudomonas fluorescens*	Microbial leaching of Fe, Ni, and Co	[178]
16	*Pseudomonas putida*	Dissolution of aluminum from metakaolin	[179]
17	*Rhodococcus* spp.	Microbial leaching of sulfur, iron, and silica	[180]
18	*Shewanella piezotolerans*	Reduction and biomineralization of iron	[181]
19	*Sporosarcina pasteurii*	Induced calcium carbonate mineralization	[182]
20	*Sulfolobus metallicus*	Copper leaching	[183]

Microbial leaching, also known as hydrometallurgical technology, utilizes the metabolic characteristics of specific microorganisms to oxidize or reduce target metal components in ores, enabling their separation in ionic or precipitated forms. This process aims to enrich valuable components or remove harmful elements. Currently, extensive research focuses on *Acidithiobacillus ferrooxidans*, which are proven to leach metals such as copper [164], zinc [184], cadmium, nickel [185], vanadium [186], uranium [187], lithium [188], phosphorus [189], iron [190], cobalt [191], molybdenum [192], and tellurium [193]. Additionally, *Paenibacillus mucilaginosus*, as a silicate-dissolving bacterium demonstrates significant desilication capabilities [37]. Ammonia-producing bacteria (e.g., *Providencia*) [194] can also leach copper. Moreover, certain substances can enhance the bioleaching process, such as surfactants [195], o-phenylenediamine [196], silver nitrate [197], and some metal ions [198]. Microbial leaching induces crystalline lattice disintegration, resulting in macroscopic mineral dissolution. This process enhances the permeability of the reservoir rock matrix while reducing flow resistance, thereby facilitating MEOR. Furthermore, ions leached from minerals stimulate the metabolic activity of other MEOR-functional microorganisms (e.g., hydrocarbon-degrading microbes and biosurfactant-producing bacteria). This auxiliary effect enables synergistic cooperation between mineral-leaching microorganisms and other MEOR-functional microorganisms.

## 9. Concluding Remarks

Although research on MEOR remains active, studies targeting minerals—both in the laboratory and field trials—are relatively inadequate (Table 1). This may not stem from a lack of insight into the full complexity of the interacting systems but rather reflect a pragmatic trade-off due to the significant mismatch between the reaction rates of microbe–mineral interactions and the scale required for oil and gas production. The cases and mechanisms reviewed here suggest that, despite its current lack of attention, mineral-targeted MEOR holds considerable promise.

Overall, the microbial inhibition of clay swelling does not reverse already-swollen clay structures, limiting its applicability in reservoirs requiring enhanced oil recovery after water flooding. This mechanism is more suitable during early-stage water injection. Both acid-producing and non-acid-producing microbes (e.g., silicate-weathering microorganisms) can significantly enhance reservoir porosity and permeability through microbial weathering. However, these mechanisms still require validation via field tests. To date, microbially induced precipitation is the only mechanism that has been field-tested—*Sporosarcina pasteurii* has successfully induced precipitation to block high-permeability channels, reduce ineffective water cycling, and improve sweep efficiency. Meanwhile, microbes that produce biosurfactants or biosolvents primarily function by reducing interfacial tension and improving displacement efficiency, which clearly falls under fluid–microbe interactions. Altering mineral surface wettability is only a secondary effect. Moreover, research on microorganisms that preferentially colonize the oil–rock interface rather than the oil–water interface remains insufficient.

Significant knowledge gaps persist in the field of mineral-targeted MEOR. Laboratory studies on microbe–mineral interactions under MEOR conditions are limited, not to mention field applications. The current understanding of such interactions is largely based on near-surface environmental conditions; their stability and effectiveness under reservoir conditions—high-temperature, high-pressure, insufficient oxygen, and hydrocarbon-rich settings—remain largely unknown.

Looking forward, microorganisms capable of efficient mineral interactions—whether through microbial mineralization, weathering, or other mechanisms—should be actively investigated for MEOR applications. Examples include silicate-degrading microbes, acid-producing microorganisms, those used in bioleaching, and those efficient at forming biominerals. With advances in petroleum engineering and genetic engineering, previous constraints on MEOR implementation—such as reservoir redox conditions, temperature, and pressure tolerance—have become less restrictive, opening new pathways for the microbial enhancement of oil recovery. We posit that incorporating microbe–mineral interaction studies into the FMM ternary system will propel MEOR into a transformative new phase.

## Data Availability

No new data were created or analyzed in this study.

## Supplementary Materials

The following supporting information can be downloaded at: https://www.mdpi.com/article/10.3390/microorganisms13122706/s1, Figure S1: Mechanism of urease-induced carbonate precipitation for plugging.

## Abbreviations

The following abbreviations are used in this manuscript:

MEOR	Microbial Enhanced Oil Recovery
FMM	Fluid–Microbe–Mineral
DIRB	Dissimilatory Iron-Reducing Bacteria
MICP	Microbially Induced Carbonate Precipitation
SARA	Saturated hydrocarbons, Aromatic hydrocarbons, Resins, and Asphaltenes
