# Supplementary material for: Mineral-Targeted Microbial Enhanced Oil Recovery
Source: Microorganisms. 2025 Nov 27;13(12):2706. doi: 10.3390/microorganisms13122706 (PMC12735141; doi:10.3390/microorganisms13122706)
Supplement: Supplementary file 1 [file microorganisms-13-02706-s001.zip › microorganisms-3889333-supplementary.pdf]

Mineral-Targeted Microbial-Enhanced Oil Recovery

Lei Li*, Chunhui Zhang, and Peidong Su

School of Chemical & Environmental Engineering, China University of Mining & Technology (Beijing), Beijing 100083, China

***Correspondence: lileilunwen@126.com**

Supplementary Materials

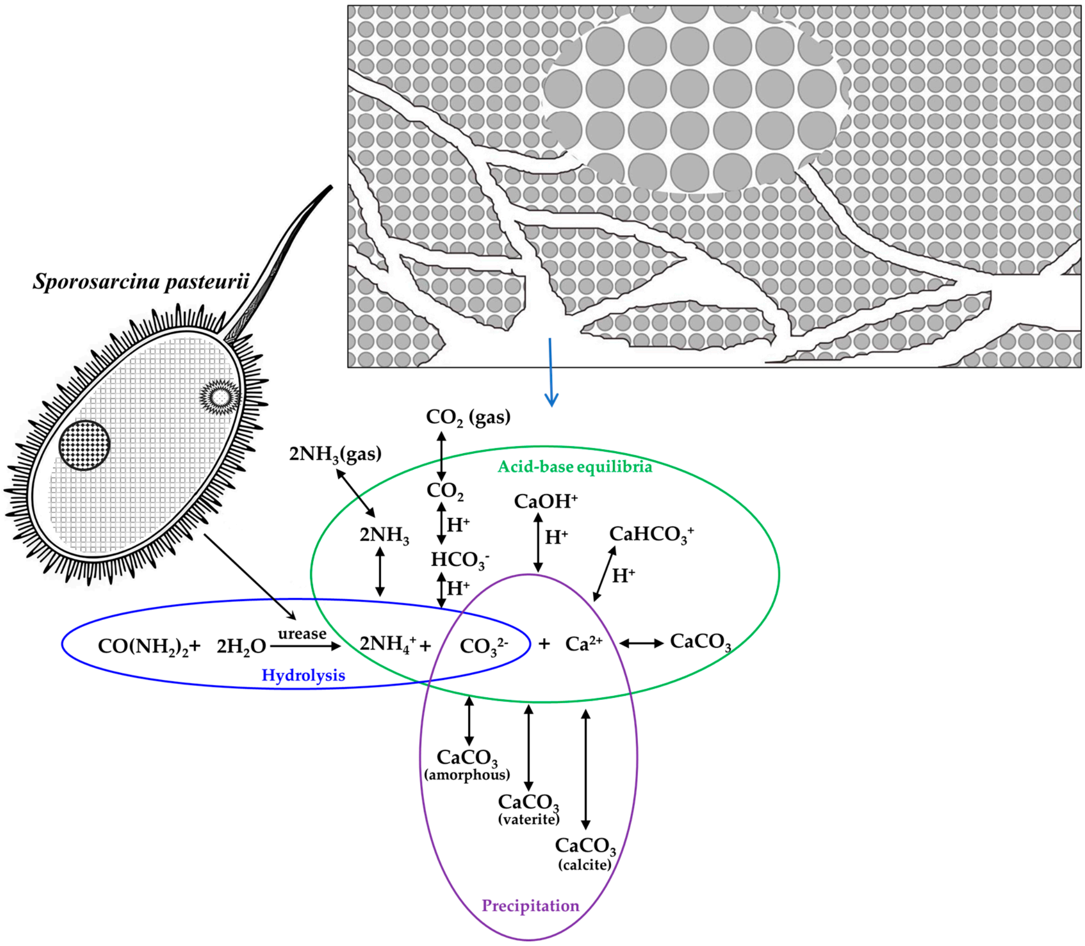

Figure S1 Mechanism of urease-induced carbonate precipitation for plugging (Reproduced according to the Ref. [35,83,95,97]).
